# Atypical Presentation of Multiple Foreign Body Ingestion

**DOI:** 10.21699/ajcr.v8i1.525

**Published:** 2017-01-05

**Authors:** Emrah Aydın, Sultan Bent, Burak Ayan

**Affiliations:** 1 Pediatric Surgery Department, Bahcelievler State Hospital, Istanbul, Turkey; 2 Department of Pediatrics, Bagcilar Education and Training Hospital, Istanbul, Turkey; 3 Anesthesiology Department, Bahcelievler State Hospital, Istanbul, Turkey

**Keywords:** Intestinal perforation, Foreign body, Children, Pica syndrome

## Abstract

Foreign body ingestion is very common in childhood especially under 3 year of age. Pica syndrome is characterized by an appetite for substances that are largely non-nutritive. We present a 3-year old girl who presented to ER with symptoms and signs of intestinal obstruction.

## CASE REPORT

A 3-year-old girl admitted to our emergency department with abdominal pain and vomiting for the last three hours. She had history of dysphagia for one month and contrast-swallow (performed a month before presentation) was found to be normal. On examination, she had abdominal distention and tenderness with no bowel sounds. Nasogastric intubation was performed. All laboratory examinations were normal including leucocyte count, CRP and biochemical analysis. Abdominal x-ray showed a radio-opaque mass located below the liver (Fig.1). Ultrasonography suspected abscess formation (33mm x 27mm) on right side of the abdomen. On CT scan there was suspicion of perforation or possible abdominal mass filling right side of abdomen. Decision of surgery was thus taken. During intubation, a button of pant was found stuck in her nostrils. Bronchoscopy was performed for other possible foreign body and infectious mucus was aspirated from the main bronchi. On exploration, intestinal perforation was found with fecal content protruding through extensively dilated ileocecal region and a mass was seen arising from it (Fig.2). Resection of terminal ileum, cecum and ascending colon was performed; and ileostomy and colonic mucous fistula were made. Postoperative recovery was uneventful. Pathology was reported as feces containing many foreign materials such as hair, plastic and some unidentified materials. She was referred to pediatric psychiatry. Four months after the operation, closure of the stoma was performed.

**Figure:**
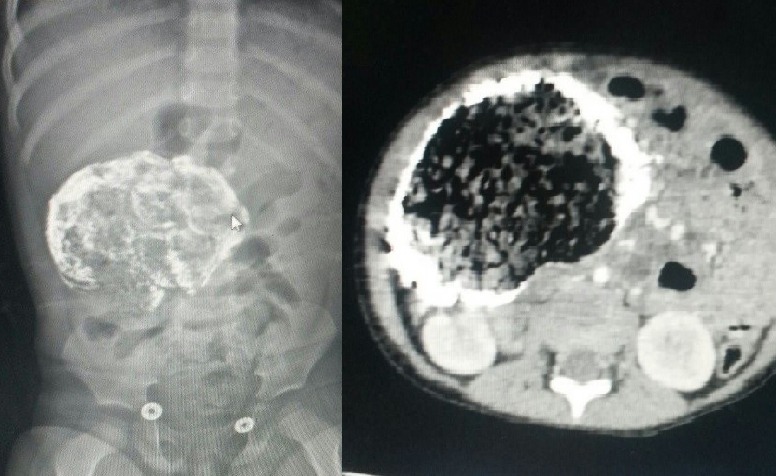
Figure 1: Contrast enhanced mass on X-ray and CT scan

**Figure:**
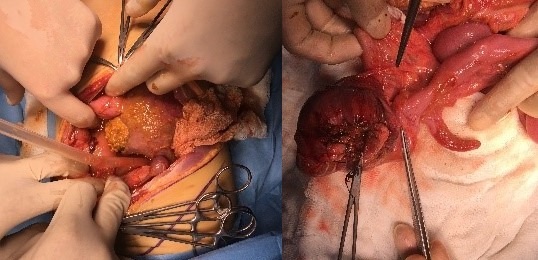
Figure 2: Peroperative view of the perforation and fecal / inflammatory mass.

## DISCUSSION

Foreign body ingestion is the second most common endoscopic emergency after GI bleeding.[1] Upper end of the esophagus is the most common site of foreign body impaction accounting over 75% of all cases.[2] In our case, there was history of difficulty in swallowing which lasted for some days. We believe, at that time she might have ingested a foreign body which was stuck at the physiologic constrictions of the esophagus. The upper contrast series was found normal as the foreign body might have moved down to the stomach or intestine. Even less than 1% perforation is a risk after foreign body ingestion. It mostly occurs at physiological sphincters such as pylorus, ileocecal valve, angulations such as duodenum, hepatic/splenic flexures, anastomosis of former operations or regions of congenital malformation.[2-4] In our case, patient had eaten many non-food items which were stuck at ileocecal region causing inflammation and perforation. We believe, the same foreign body which was stuck at esophagus might have caused perforation a month before presentation as the contrast material (contrast study performed at that time) was found in the fecal mass containing many foreign bodies.

## Footnotes

**Source of Support:** Nil

**Conflict of Interest:** None declared

